# CPR training initiative: a novel training approach of nursing health care staff in CPR/ACLS, for quality improvement in CPR in India – an observational study

**DOI:** 10.1186/1865-1380-7-S1-P3

**Published:** 2014-07-25

**Authors:** Saravana Kumar, Syed Ahmed Adil, Benny Remi Ponnappan, Anindya Das Gupta

**Affiliations:** 1 Mehta Group of hospitals, Chetpet, Chennai, Tamilnadu, India

## Objective(s)

In India, Advanced Cardiovascular Life Support (ACLS) certification is not part of routine training during medical or nursing schooling and such trainings are offered in only very few centers during selected days with limited participant numbers. It is not possible to enroll all health care staff in the hospital for such a training as it is time consuming and the cost of such training programs is high. Training in trauma and cardiac life support and certification in both adult and pediatric, would easily cost more than 22,000 Indian rupees and it is a significant cost for staff to afford it. The cheapest way would be to resort to in-house training of their health care staffs in ACLS and CPR by in-house qualified ACLS trainers with available logistics like mannequins and other equipment.

Many research studies have evaluated knowledge of CPR among various groups of health care providers; however none of the studies that we are aware of in India have re-assessed the knowledge after training such a study group or suggested a novel training approach and duration for such in house training.

This study was undertaken to observe and suggest an easier approach for training all the in-house staffs in ACLS as the logistics are limited in most of our Indian setups and try to define a duration and criteria if possible for an effective training to those institutions whose resources are limited.

## Methods

A step wise approach for assessment, training and re assessment was carried out as per the protocol prepared by the department of Accident and Emergency Medicine in our multi-specialty hospital. Among 160 nursing staff members who were posted in the non-critical areas of the hospital, 42 members were chosen by random sampling and a surprise scenario based ACLS mock assessment with mannequin was conducted in various areas of the hospital included in the study over a period of four days during various shift timings. The initial response of the nursing staff members during early few critical minutes of a cardiopulmonary resuscitation was assessed and recorded in the checklist prepared by the emergency department focusing on the critical steps of initial five minutes in a resuscitation. Clinical training of a two hour duration was done based on AHA protocol for all the nursing in charges of the wards who are potential instructors, by the Department of Accident and Emergency Medicine team. They were evaluated and certified to be in-house trainers. An in house “CPR Instructor” group was formed. The task of training other staff in a period of two weeks was set to these “in-house CPR Instructor group”. All other 160 staff of the non-critical area of the hospital where grouped under these 14 hospital certified CPR instructors and hands on training was given to them in their respective wards by these certified trainers whenever they could find time. Two adult CPR mannequins were kept available 24x7 for training purpose.

## Results

Statistically significant improvement in the compliance and performance of CPR / ACLS was noted after the training done in-house.

## Conclusion

A simple in-house training approach can significantly improve the knowledge and performance of our health care staff in ACLS and CPR and can be repeated whenever needed by creating more in-house trainers. Expensive training and expensive recertification from outside associations is not needed for all.

